# PathMED: an R toolkit for single-sample molecular scoring and machine learning with omics data

**DOI:** 10.1093/bioinformatics/btag519

**Published:** 2026-07-24

**Authors:** Jordi Martorell-Marugán, Ivan Ellson, Raúl López-Domínguez, Pablo Pedro Jurado-Bascón, Juan Antonio Villatoro-García, Chang Wang, Frédéric Baribaud, Daniel Toro-Domínguez, Pedro Carmona-Sáez

**Affiliations:** Computational Neuroscience, Joint Unit in Biomedical Imaging and Artificial Intelligence FISABIO–CIPF, Foundation for the Promotion of Health and Biomedical Research in the Valencian Region (FISABIO), Valencia 46012, Spain; Bioinformatics and Health Data Science, GENYO, Centre for Genomics and Oncological Research: Pfizer, University of Granada, Andalusian Regional Government, PTS Granada, Granada 18016, Spain; Fundación para la Investigación Biosanitaria de Andalucía Oriental-Alejandro Otero (FIBAO), Granada 18012, Spain; Bioinformatics and Health Data Science, GENYO, Centre for Genomics and Oncological Research: Pfizer, University of Granada, Andalusian Regional Government, PTS Granada, Granada 18016, Spain; Bioinformatics and Health Data Science, GENYO, Centre for Genomics and Oncological Research: Pfizer, University of Granada, Andalusian Regional Government, PTS Granada, Granada 18016, Spain; Bioinformatics and Health Data Science, GENYO, Centre for Genomics and Oncological Research: Pfizer, University of Granada, Andalusian Regional Government, PTS Granada, Granada 18016, Spain; Department of Statistics and Operational Research, University of Granada, Granada 18071, Spain; Bioinformatics and Health Data Science, GENYO, Centre for Genomics and Oncological Research: Pfizer, University of Granada, Andalusian Regional Government, PTS Granada, Granada 18016, Spain; Department of Statistics and Operational Research, University of Granada, Granada 18071, Spain; Translational Early Program Lead, ICV Translational Early Development, Bristol Myers Squibb, Lawrence, NJ 08543, United States; Translational Early Program Lead, ICV Translational Early Development, Bristol Myers Squibb, Lawrence, NJ 08543, United States; Department of Environmental Medicine, Karolinska Institutet, Unit of Inflammatory Diseases, Solna, Sweden; Bioinformatics and Health Data Science, GENYO, Centre for Genomics and Oncological Research: Pfizer, University of Granada, Andalusian Regional Government, PTS Granada, Granada 18016, Spain; Department of Statistics and Operational Research, University of Granada, Granada 18071, Spain

## Abstract

**Motivation:**

Molecular scoring is a popular approach for studying pathway-level functional alterations with omics data. Using molecular scores for tasks such as single-sample molecular characterisation, phenotype prediction or disease stratification has several advantages compared to using omics data directly. Molecular scores provide biological interpretability and are more generalisable across datasets, facilitating data integration and machine learning applications. However, numerous scoring methods are available through different software packages, and currently there is a lack of tools to easily use these scores for model training and prediction.

**Results:**

We developed pathMED, an R/Bioconductor package that unifies various scoring methods in a simple framework. Furthermore, pathMED also contains a machine learning module to train and test models that use the calculated molecular scores to predict clinical outcomes. We demonstrate some of its potential applications in three use cases using public omics data. We showed the generalisability of machine learning models trained on transcriptomic scores in predicting clinical outcomes when deploying on proteomic scores. We also demonstrated the application of transcriptomics scores in predicting breast cancer treatment response and identifying pathways strongly associated to tumour biology and treatment response. Finally, we demonstrated the benefit of integrating a novel gene set dissection step into the analysis pipeline to resolve disease heterogeneity at the pathway level.

**Availability:**

PathMED is freely available in the Bioconductor repository (https://bioconductor.org/packages/release/bioc/html/pathMED.html). Code to reproduce the analyses is publicly available at https://github.com/GENyO-BioInformatics/pathMED_article.

## 1 Introduction

Omics technologies, like transcriptomics or proteomics, have enabled advances towards the use of precision medicine for complex diseases by enabling predictions of clinical outcomes (e.g. treatment response) based on individual patient molecular profiles (Karczewski and Snyder 2018, Sammut *et al*. 2022). However, their translatability for diagnostic or prognostic purposes remains limited. A key reason is data variability across studies due to different technological platforms, protocols or processing steps, leading to reduced generalisability. For instance, gene lists analysed in RNA-Sequencing (RNA-Seq) experiments might differ due to biological variations or technical factors such as the use of different bioinformatics pipelines or the presence of batch effects. This can lead to difficulties in the analysis and comparison of molecular patterns across studies.

Single-sample molecular scoring methods address these challenges by using individual feature information (e.g. gene expression) to calculate scores for molecular signatures, typically gene sets. These molecular scores provide information about the activity of specific gene signatures that may represent different biological aspects depending on the database used. For example, Gene Ontology (GO) (Ashburner *et al*. 2000, Aleksander *et al.* 2023) contains gene sets for biological processes, cellular components and molecular functions, while the Kyoto Encyclopedia of Genes and Genomes (KEGG) (Kanehisa and Goto 2000) characterises biological systems by pathways. Molecular scores also enhance interpretability and are more generalisable across studies than gene-level data, as it has been demonstrated that variations across individual datasets are mitigated by transforming genes into a common functional space (Toro-Domínguez *et al*. 2025). Furthermore, molecular scores are more robust to confounding factors than feature-level data, and thus are better suited for generalisable predictive modelling, consistent patient stratifications and the study of disease heterogeneity (Pavlidis *et al*. 2019a, Toro-Domínguez *et al*. 2022, Hubbard *et al*. 2023).

Several techniques have been proposed for single-sample molecular scoring, including single-sample Gene Set Enrichment Analysis (ssGSEA) (Barbie *et al*. 2009), Gene Set Variation Analysis (GSVA) (Hänzelmann *et al*. 2013) or singscore (Foroutan *et al*. 2018), among others. We also recently developed M-scores, a novel method that allowed us to evaluate the pathological mechanisms of individual Systemic Lupus Erythematosus (SLE) patients and to predict drug response, symptoms and disease progression (Toro-Domínguez *et al*. 2022).

A common limitation of molecular scoring systems is that gene signatures often comprise broad groups of features that may exhibit heterogeneous behaviours (Ackermann and Strimmer 2009). For example, only a subset of genes belonging to a given pathway might show coexpression patterns in a particular study, and this subset of genes may vary across different study conditions (Hejblum *et al*. 2015). Moreover, different genes annotated with the same pathway might exhibit opposite patterns (i.e. some may be overexpressed while others may be underexpressed). Direct scoring of these heterogeneous signatures without accounting for such plasticity may result in a significant loss of information, as a single activity value can mask the heterogeneity. This problem has been previously reported, and some solutions have been proposed to split the signatures into small subsets with coordinated activity based on omics data (Hejblum *et al*. 2015). Furthermore, increasing the granularity of functional annotations in specific conditions can also reveal previously masked subpathways important for those conditions.

Molecular scores may also provide an alternative data representation on which machine learning (ML) models can be trained, instead of using gene-level expression. This can facilitate the comparison and integration of different datasets during both model construction and evaluation, as has been previously demonstrated in studies of SLE (Toro-Domínguez *et al*. 2022, Hubbard *et al*. 2023) and inflammatory bowel disease (Pavlidis *et al*. 2019). Another advantage of molecular scores in downstream applications is that they solve the problem of gene-level missing data across studies, as the calculation of scores does not require the presence of all the genes in each gene set (Toro-Domínguez *et al*. 2025).

Different R packages have been developed to apply the available scoring methods, including GSVA (Hänzelmann *et al*. 2013), decoupleR (Badia-I-Mompel *et al*. 2022) or singscore (Foroutan *et al*. 2018). Although useful, these packages require users to provide gene sets following specific formats, which can be time-consuming especially when different gene set databases or methods are used. Furthermore, they did not provide some additional features in the context of molecular scoring, such as splitting heterogeneous gene sets into subsets with coordinated activity, annotating gene sets identifiers to biological terms or using scores to train and validate ML models.

To unify single-sample scoring techniques into a robust framework for omics-based precision medicine, we developed the R package pathMED. We simplified molecular scoring, signatures splitting, ML model training and validation and prediction on new samples. We incorporated 20 scoring methods, 16 ML algorithms for binary, multiclass and continuous predictions, as well as other useful functionalities for using omics data in precision medicine. We tested pathMED with three use cases, demonstrating its utility to interoperate with different types of omics data, to identify relevant pathways for cancer treatment response and to reveal relevant subpathways overlooked with standard approaches.

## 2 Methods

### 2.1 Preloaded databases

Molecular scoring in pathMED can be performed using any gene set annotation provided by the user. Additionally, for convenience, the package includes 12 commonly used, preloaded databases for human genes: GO—Biological Process (GO BP), GO—Molecular Function (GO MF), GO—Cellular Component (GO CC) (Ashburner *et al*. 2000, Aleksander *et al.* 2023), KEGG (Kanehisa and Goto 2000), Reactome (Milacic *et al*. 2024), Pharmacogenomics Knowledgebase (PharmGKB) (Whirl-Carrillo *et al*. 2021), Library of Integrated Network-based Cellular Signatures (LINCS) (Keenan *et al*. 2018), Comparative Toxicogenomics Database (CTD) (Davis *et al*. 2025), DisGeNET (Piñero *et al*. 2020), Human Phenotype Ontology (HPO) (Gargano *et al*. 2024), WikiPathways (Agrawal *et al*. 2024) and blood-derived coexpression modules (Chaussabel *et al*. 2008, Li *et al*. 2014). All gene sets, except the coexpression modules, were retrieved from the GeneCodis database (Garcia-Moreno *et al*. 2022) and formatted for compatibility with pathMED. Coexpression modules were retrieved from the tmod package (Zyla *et al*. 2019).

### 2.2 Molecular signatures dissection

Most molecular signatures in public databases are primarily based on scientific literature, providing lists of features (e.g. genes) linked to biological functions. However, the activities of the features within the same signature are not expected to be synchronized. For example, some gene sets contain both activators and repressors of a biological process. This functional heterogeneity is reflected in omics data, and calculating a single molecular score for the function may hinder distinct patterns in these heterogeneous signatures.

To address this, pathMED includes the *dissectDB* function. This function decomposes gene sets into coexpressed subsets specific to the phenotype of interest, enhancing granularity and interpretability. To enable this function, users should provide one or more datasets, which can optionally include reference samples (e.g. healthy controls). If reference samples are provided, their means and standard deviations (SDs) are used to standardize expression values by gene-wise *Z*-scoring (Kim *et al*. 2018). Otherwise, traditional *Z*-scores are calculated across the case samples using their own mean and SD.

Each gene set is then clustered using k-means. The number of clusters is chosen to exceed a user-defined variance threshold. Small clusters (fewer than three features by default) are merged with their nearest cluster based on Euclidean distance, as gene sets of this size are generally insufficient to produce stable and interpretable scores. When multiple datasets are provided, the *dissectDB* function in pathMED performs the clustering in two stages: **(**1) Dataset-specific clustering: Each dataset is analysed independently, resulting in a set of preliminary gene subsets per dataset. (2) Cross-dataset alignment: For each gene set, a matrix of gene-subcluster membership is built, containing the subclusters identifiers identified in the first clustering round for each dataset. K-means clustering is applied to obtain the conserved subpathways across the different studies. This two-step procedure ensures that the resulting subsets are not driven by a single dataset but instead represent robust gene groupings that are reproducible across independent cohorts. Importantly, all subsets retain the annotation to their set of origin, meaning that signatures dissection does not imply losing information about the original databases.

### 2.3 Scoring methods

PathMED includes 20 molecular scoring methods, including widely used techniques such as GSVA (Hänzelmann *et al*. 2013), ssGSEA (Barbie *et al*. 2009) or singscore (Foroutan *et al*. 2018) (Table 1). We unified their input format and parameters into a single function, facilitating their use and interoperability. Weights may be incorporated into many of these methods (Table 1) to use known information about functional relationships among features within gene sets (e.g. transcription factor targets).

**Table 1:** Single sample scoring approaches included in pathMED.

Method	Type	Weighted network	Original package	Reference
GSVA	Rank	No	GSVA	(Hänzelmann *et al.* 2013)
ssGSEA	Rank	No	GSVA	(Barbie *et al.* 2009)
Fast Gene Set Enrichment Analysis (FGSEA)	Rank	Yes	decoupleR	(Korotkevich *et al.* 2021)
Normalized FGSEA (norm_FGSEA)	Rank	Yes	decoupleR	(Korotkevich *et al.* 2021)
singscore	Rank	No	singscore	(Foroutan *et al.* 2018)
*Z*-score	Parametric	No	GSVA	(Lee *et al.* 2008)
M-Score	Parametric	No	pathMED	(Toro-Domínguez *et al.* 2022)
Pathway Level Analysis of Gene Expression (PLAGE)	Parametric	No	GSVA	(Tomfohr *et al.* 2005)
Weighted Mean (WMEAN)	Aggregation	Yes	decoupleR	(Badia-I-Mompel *et al.* 2022)
Normalized WMEAN (norm_WMEAN)	Aggregation	Yes	decoupleR	(Badia-I-Mompel *et al.* 2022)
Corrected WMEAN (corr_WMEAN)	Aggregation	Yes	decoupleR	(Badia-I-Mompel *et al.* 2022)
Weighted Sum (WSUM)	Aggregation	Yes	decoupleR	(Badia-I-Mompel *et al.* 2022)
Normalized WSUM (norm_WSUM)	Aggregation	Yes	decoupleR	(Badia-I-Mompel *et al.* 2022)
Corrected WSUM (corr_WSUM)	Aggregation	Yes	decoupleR	(Badia-I-Mompel *et al.* 2022)
AUCell	Rank	Yes	decoupleR	(Aibar *et al.* 2017)
Over Representation Analysis (ORA)	Fisher’s exact test	Yes	decoupleR	(Badia-I-Mompel *et al.* 2022)
Univariate Decision Tree (UDT)	Decision trees	Yes	decoupleR	(Badia-I-Mompel *et al.* 2022)
Multivariate Decision Trees (MDT)	Decision trees	Yes	decoupleR	(Badia-I-Mompel *et al.* 2022)
Univariate Linear Model (ULM)	Linear models	Yes	decoupleR	(Teschendorff and Wang 2020)
Multivariate Linear Model (MLM)	Linear models	Yes	decoupleR	(Badia-I-Mompel *et al.* 2022)

Additionally, the framework includes *M*-Score, a novel scoring method developed by our group, which was successfully applied in the context of SLE (Toro-Domínguez *et al*. 2022). The M-Score for a given sample *s* and gene set *gs* (*M-Score_sgs_*) is calculated following Equation 1:


(1) $${M-\mathrm{Score}}_{\mathrm{sgs}}=\frac{\sum_{j=1}^{n_{i}}\left(\frac{x_{j}-\mu_{jH}}{\sigma_{jH}}\right)}{n_{i}}$$

*x_j_* being the expression of gene *j* in each sample, *μ_jH_* and *σ_jH_* being the mean and standard deviations of gene *j* in reference samples respectively, and *n_i_* the number of members in the gene set.

Although the *M*-Score has the advantage of incorporating deviations from reference samples (e.g. healthy controls), the original function requires the presence of reference samples in the user-provided dataset for analysis, which limits its application. To overcome this limitation, pathMED includes a set of functions to create a reference from several datasets (including disease samples and healthy controls), making it possible to use this reference to impute *M*-scores for new datasets without healthy samples (Toro-Domínguez *et al*. 2022) (Fig. S1, available as supplementary data at *Bioinformatics* online). Briefly, score imputation for new patients is based on Euclidean similarity of gene expression profiles to reference samples with previously calculated M-scores.

### 2.4 Machine learning framework

PathMED includes a tuneable supervised ML framework applicable to any data matrix, including molecular scores and feature-level omics data. The framework employs nested cross-validation (nCV), with user-defined inner and outer fold numbers. In each outer cross-validation (CV) iteration, the training set is used to optimize the model hyperparameters through an inner repeated CV. The resulting optimized model then predicts the test set labels. Performance metrics, averaged across all outer nCV folds, are reported.

Users can select one or multiple ML algorithms (Table 2) for the analysis, reporting performance metrics for each one. Furthermore, a greedy ensemble of the included ML models is adjusted. The best-performing algorithm based on the metric chosen by the user (by default, Matthew’s correlation coefficient (MCC) for classification and correlation for regression) is used to train a final model with the complete dataset, using the nCV-tuned optimal hyperparameters. This final model is exported for use with external data. If there are non-independent samples (e.g. replicates for the same individual), it can be indicated to the function, so they are always included in the same set and avoid data leakage. All ML methods are implemented using the caret package (Kuhn 2008).

**Table 2:** ML models included in pathMED.

Algorithm	Name in PathMED	Type	Categorical/continuous	Linear/non-Linear	Reference
Generalized Linear Model	glm	Linear model	Both	Linear	(McCullagh 1989)
Linear Regression	lm	Linear model	Continuous	Linear	(Kenney and Keeping 1962)
Linear Discriminant Analysis	lda	Discriminant analysis	Categorical	Linear	(Cox 1958)
eXtreme Gradient Boosting	xgbTree	Tree-based	Both	Non-linear	(Chen and Guestrin 2016)
Random Forest	rf	Tree-based	Both	Non-linear	(Breiman 2001)
k-Nearest Neighbors	knn	Instance-based	Both	Non-linear	(Mucherino *et al.* 2009)
Support Vector Machine with Linear Kernel	svmLinear	Support vector machine	Both	Linear	(Cortes and Vapnik 1995)
Single-hidden-layer Neural Network	nnet	Neural network	Both	Non-linear	(Ripley 1996)
Support Vector Machines with Radial Basis Function Kernel	svmRadial	Support vector machine	Both	Non-linear	(Cortes and Vapnik 1995)
Naive Bayes	nb	Bayesian model	Categorical	Linear	(Zhang 2004)
Least Angle Regression	lars	Linear model	Continuous	Linear	(Efron *et al.* 2004)
Classification and Regression Tree	rpart	Tree-based	Both	Non-linear	(Breiman *et al.* 1984)
Boosted Generalized Additive Model	gamboost	Additive model	Both	Non-linear	(Tutz and Binder 2006)
Boosted Classification Trees	ada	Tree-based	Categorical	Non-linear	(Schapire 2013)
Bayesian Regularized Neural Networks	brnn	Neural network	Continuous	Non-linear	(MacKay 1992, Dan Foresee and Hagan 1997)
Elasticnet	enet	Linear model	Continuous	Linear	(Zou and Hastie 2005)

### 2.5 Multi-omics use case

To demonstrate the generalisability of molecular scores across data types, we used transcriptomics and proteomics data from paired tumour and normal tissue samples from 110 lung adenocarcinoma (LUAD) patients. Data was retrieved from the Clinical Proteomic Tumor Analysis Consortium (CPTAC) through the LinkedOmicsKB resource (https://kb.linkedomics.org/). For transcriptomics, we filtered out genes located in pseudoautosomal regions. For both omics data types, we converted ENSEMBL identifiers to gene symbols, aggregating the gene symbols with more than one ENSEMBL identifier by their median. In total, 40 927 and 7899 features remained from transcriptomics and proteomics data respectively.

We used pathMED to calculate scores for GO BP gene sets with each omic type using most of the scoring methods available in the package. As the available genes in each omic type differ, not all the gene sets in GO BP could be scored in both data types. Thus, we subset the data to include 9,731 gene sets shared by both.

We randomly divided the patients into a training set (75%) and a test set (25%). Using the transcriptomic data from the training set patients, we trained Support Vector Machine (SVM) models with a linear kernel. Model training and hyperparameter optimization were performed using the nCV implemented in pathMED, with five outer folds and four inner folds. The models were trained to classify samples as either tumour or normal tissue. Using the pathMED functionalities, we considered the patient identifiers during the nCV folds design to always include normal and tumour samples from the same patients in the same subsets. Then, we used the exported trained model to predict the labels on the test set patients using their corresponding proteomics data. This entire process was repeated 10 times to avoid potential biases in the initial train-test split.

Additionally, we used the model trained with the whole transcriptomics dataset to predict the labels from the 359 proteomics samples of the Taiwan Cancer Moonshot (Chen *et al*. 2020), previously processed (Satpathy *et al*. 2025).

### 2.6 Breast cancer use case

To demonstrate the potential of the pathMED framework predicting complex clinical variables, we applied it to predict treatment response of breast cancer patients using whole blood RNA-Seq data from 149 patients collected prior to neoadjuvant treatments in a previous study (Sammut *et al*. 2022). Treatment response was classified as pathological complete response (pCR) or residual disease (RD), with RD grade estimated with the residual cancer burden (RCB) score. Among the 149 patients in this study, 39 patients achieved pCR post-treatment, while the remaining 110 had RD with RCB scores ranging from 0.558 to 4.255. The patients with RD were further classified as having minimal (RCB-I, *n = *23), moderate (RCB-II, *n = *62) or extensive residual disease (RCB-III, *n = *25).

We normalized raw counts using the trimmed mean of *M*-values (TMM) method (Robinson and Oshlack 2010) implemented in the edgeR package (Chen *et al*. 2025). Normalized counts were log2-transformed. We then used the GSVA method to score gene sets from GO BP, GO MF, Reactome and KEGG. For each database, we then selected the 1000 gene sets with the highest variance in this dataset as input features for the ML models.

Using the computed scores, we predicted the binary pCR/RD outcome with Linear Discriminant Analysis (LDA) and the continuous RCB score with Random Forest (RF). For both tasks, we used the nCV implemented in pathMED with three inner and outer folds.

The importance of gene sets in the trained models was estimated with the caret package. For the binary prediction with LDA, the absolute coefficients from the discriminant function were retrieved. For the regression with RF, the percentage increase in the mean squared error after permuting was obtained. Both metrics were used to rank the gene sets according to their relative importance in the models. We annotated the gene sets terms with the *ann2term* function available in pathMED.

### 2.7 Ulcerative colitis use case

In this use case, we aimed to demonstrate the utility of splitting heterogeneous gene sets into more homogeneous subsets with coordinated activity. We used gene expression measured with the Affymetrix Human Genome U219 Array sigmoid colonic biopsies from 57 ulcerative colitis (UC) patients and 19 healthy controls (two samples per subject) from a previous study (Soendergaard *et al*. 2017). Processed data is available in NCBI-GEO (identifier: GSE206171). We calculated molecular scores with all available scoring methods in PathMED using the original Reactome pathways, as well as the coexpressed subpathways defined by the *dissectDB* function. We calculated the Pearson’s correlation between molecular scores and the severity metric Mayo score (Schroeder *et al.* 1987). Scoring methods with an absolute mean correlation in the top 100 pathways lower than 0.6 were discarded.

We estimated the capacity to predict Mayo score with both the original and dissected databases applying the pathMED training and validation pipeline with RF, *k*-Nearest Neighbors (KNN) and SVM algorithms. The prediction accuracy of this continuous variable was estimated with the Pearson’s correlation coefficient (*r*), Root Mean Square Error (RMSE), *R*-Squared (*R*^2^), Mean Absolute Error (MAE), relative MAE (RMAE) and Relative Standard Error (RSE) metrics.

## 3 Results and discussion

### 3.1 pathMED R package

**Figure 1:**
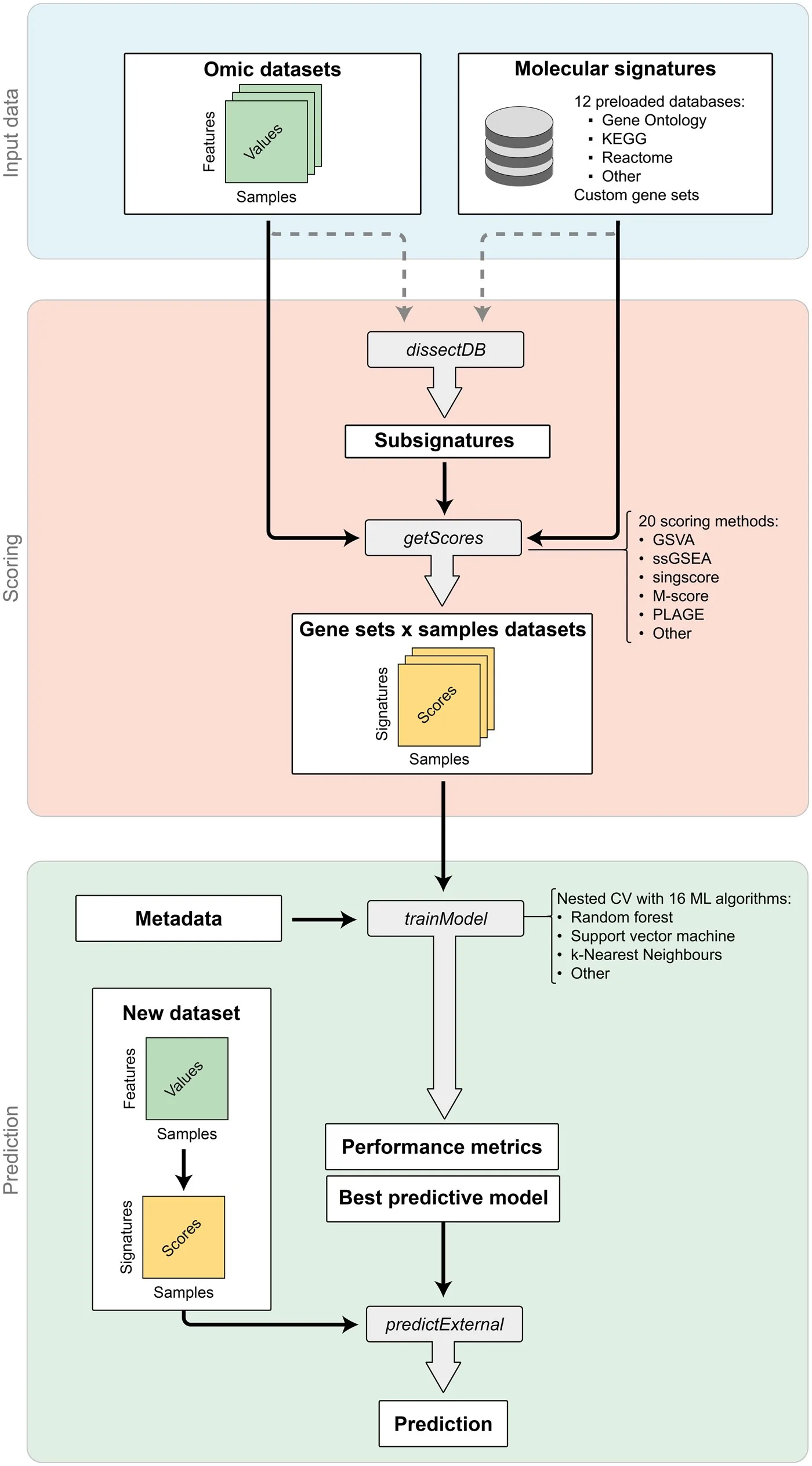
Workflow with the main pathMED functions. Gray boxes indicate package functions. Input data consists of omic datasets and molecular signatures databases, which may be provided or chosen from the prebuilt ones. In the scoring step, signatures may optionally be split into subsignatures for scoring, or scores may be calculated directly on the original signatures. In the training step, metadata is used to train and evaluate ML models in a nCV, resulting in performance metrics and an exported model that may be used to make predictions on new external data following molecular scoring.

PathMED is a comprehensive framework that integrates multiple molecular signature scoring methods with ML to characterise the molecular landscape of individual patients and predict clinical outcomes. The package contains 12 preloaded databases to streamline molecular scoring with 20 included methods. Importantly, it includes a function to split heterogeneous gene sets into subsets with coordinated activity based on the input molecular data. Figure 1 illustrates the pipeline with the included functions in the package.

### 3.2 Molecular scores are generalisable across omics data types

In this first use case, we aimed to test whether transforming related omic data types into a common gene set space enables predictive model training with one data type and predicting with another one. To address this, we used public transcriptomics and proteomics data from LUAD tumour and normal tissue samples, scored with the GO BP gene sets. We used all the scoring methods implemented in pathMED, except for *M*-Scores because it uses the control samples for score calculation and compared their performance in predicting tumour and normal tissue types (Table 3).

**Table 3:** Performance metrics of the prediction of tumour/normal tissue samples using multi-omics data.

Method	Accuracy	Precision	Recall	Specificity	BalAcc	*F*-score	MCC	NPV
GSVA	1 (0.02)	1 (0.02)	1 (0.02)	1 (0.02)	1 (0.02)	1 (0.02)	1 (0.03)	1 (0.02)
*Z*-score	1 (0.05)	1 (0.03)	1 (0.07)	1 (0.09)	1 (0.05)	1 (0.04)	1 (0.13)	1 (0.15)
ssGSEA	1 (0.02)	1 (0.02)	1 (0.02)	1 (0.02)	1 (0.02)	1 (0.02)	1 (0.04)	1 (0.02)
singscore	1 (0.04)	1 (0.03)	1 (0.06)	1 (0.05)	1 (0.04)	1 (0.04)	1 (0.13)	1 (0.18)
Plage	0.92 (0.07)	0.87 (0.07)	1 (0.1)	0.82 (0.07)	0.91 (0.07)	0.93 (0.08)	0.84 (0.15)	1 (0.08)
UDT	0.53 (0.02)	0.54 (0.02)	0.56 (0.02)	0.49 (0.02)	0.53 (0.02)	0.55 (0.02)	0.06 (0.03)	0.51 (0.02)
MDT	0.53 (0.02)	0.53 (0.02)	0.84 (0.02)	0.2 (0.02)	0.52 (0.02)	0.65 (0.02)	0.04 (0.04)	0.52 (0.02)
WSUM	0.48 (0.02)	0.5 (0.02)	0.53 (0.03)	0.44 (0.02)	0.48 (0.02)	0.51 (0.03)	−0.03 (0.04)	0.46 (0.02)
MLM	0.48 (0.02)	0.5 (0.02)	0.51 (0.02)	0.45 (0.02)	0.48 (0.02)	0.51 (0.02)	−0.04 (0.03)	0.46 (0.02)
corr_WMEAN	0.48 (0.02)	0.49 (0.02)	0.48 (0.02)	0.47 (0.02)	0.47 (0.02)	0.49 (0.02)	−0.05 (0.03)	0.46 (0.02)
AUCell	0.47 (0)	0.49 (0)	0.5 (0)	0.45 (0)	0.47 (0)	0.49 (0)	−0.06 (0)	0.45 (0)
corr_WSUM	0.47 (0.01)	0.49 (0)	0.48 (0.01)	0.46 (0)	0.47 (0.01)	0.48 (0.01)	−0.06 (0.01)	0.45 (0.01)
ULM	0.47 (0)	0.49 (0)	0.49 (0)	0.45 (0)	0.47 (0)	0.49 (0)	−0.06 (0)	0.45 (0)
WMEAN	0.47 (0.06)	0.49 (0.08)	0.49 (0)	0.45 (0.12)	0.47 (0.06)	0.49 (0.05)	−0.06 (0.1)	0.45 (0)
norm_WMEAN	0.47 (0.01)	0.49 (0)	0.49 (0.01)	0.45 (0)	0.47 (0.01)	0.49 (0.01)	−0.06 (0.01)	0.45 (0.01)
norm_WSUM	0.47 (0.02)	0.49 (0.02)	0.49 (0.03)	0.45 (0.03)	0.47 (0.02)	0.49 (0.03)	−0.06 (0.05)	0.45 (0.02)
FGSEA	0.47 (0.02)	0.49 (0.02)	0.49 (0.04)	0.45 (0.03)	0.47 (0.02)	0.49 (0.03)	−0.07 (0.05)	0.45 (0.03)
norm_FGSEA	0.47 (0.02)	0.48 (0.02)	0.48 (0.02)	0.45 (0.02)	0.46 (0.02)	0.48 (0.02)	−0.07 (0.03)	0.44 (0.02)
ORA	0.47 (0.02)	0.49 (0.02)	0.83 (0.03)	0.08 (0.02)	0.45 (0.02)	0.62 (0.02)	−0.14 (0.04)	0.29 (0.02)

BalAcc, balanced accuracy; MCC, Matthew’s correlation coefficient; NPV, negative predictive value.

Values correspond to the mean across 10 iterations and the standard deviation.

SVM models using scores from GSVA, *Z*-score, ssGSEA, singscore and Plage methods achieved outstanding performance in sample classification, achieving almost perfect accuracy rates across metrics (Table 3). However, the rest of the scoring methods failed to generalise between omic data types, as most failed to demonstrate an accuracy exceeding that of random expectation (i.e. 0.5). Together, these results demonstrate the interoperability among different omics data types by transforming gene-level matrices from different data types to a common space of gene sets. However, it is worth noting that not all the scoring methods perform equally well in this task. This observation is consistent with our recent report of benchmarking molecular scoring methods, where we identified the same methods to be more generalisable across studies (Toro-Domínguez *et al*. 2025). Nevertheless, to assess the generalisability of the trained models with transcriptomics data, we predicted the labels from an independent cohort with different demographic characteristics (Chen *et al*. 2020) using proteomics tumour and normal samples. We obtained an accuracy of 0.97 and a MCC of 0.95, indicating that this inter-omics operability is robust even across very different cohorts.

We then explored whether using scores calculated from different gene set databases could result in different predictive performances. We re-ran the analysis using all 12 preloaded databases in pathMED using the Z-Scores method, which is among the top-performing methods discussed above. The results indicated that accurate predictions are achievable using any of the 12 databases (Table S1, available as supplementary data at *Bioinformatics* online), suggesting no significant differences among tested gene set databases for prediction.

### 3.3 PathMED identifies pathways associated with breast cancer immunotherapy response

In the second use case, we aimed to predict neoadjuvant treatment response using pre-treatment RNA-Seq data from whole blood samples of 149 breast cancer patients. Briefly, GSVA scores calculated with GO BP, GO MF, Reactome and KEGG databases separately were used to predict treatment response that was defined as a binary outcome (i.e. pCR or RD) or a continuous RCB score. We observed that the performance of predicting either the binary outcome or the continuous RCB score was moderate, with subtle differences using different databases (Tables S2 and S3, available as supplementary data at *Bioinformatics* online). For models predicting the binary outcome, a consistent trend of high accuracy and specificity but relatively low precision was detected when using different databases (Table S2, available as supplementary data at *Bioinformatics* online). For example, using the GO BP database, the model achieved the highest accuracy (77.90%) among all four databases with a specificity of 0.8634 (Table S2, available as supplementary data at *Bioinformatics* online), indicating strong performance of predicting non-response to treatment. In contrast, the precision was only 0.5922 (Table S2, available as supplementary data at *Bioinformatics* online), suggesting a high rate of false positives (patients predicted to respond but who did not). For models predicting the continuous RCB score, their performance was similar when evaluated by the mean absolute error (MAE), with the lowest mean MAE at 0.9516 was detected when using the GO MF database (Table S3, available as supplementary data at *Bioinformatics* online). The observed moderate performance can potentially be attributed to the use of transcriptomics data alone, while many other clinical and molecular factors may also play an important role in predicting the complex treatment response. In fact, the original study combined transcriptomics with clinical, DNA sequencing and digital phenotype data to yield reliable predictions (Sammut *et al*. 2022).

**Figure 2:**
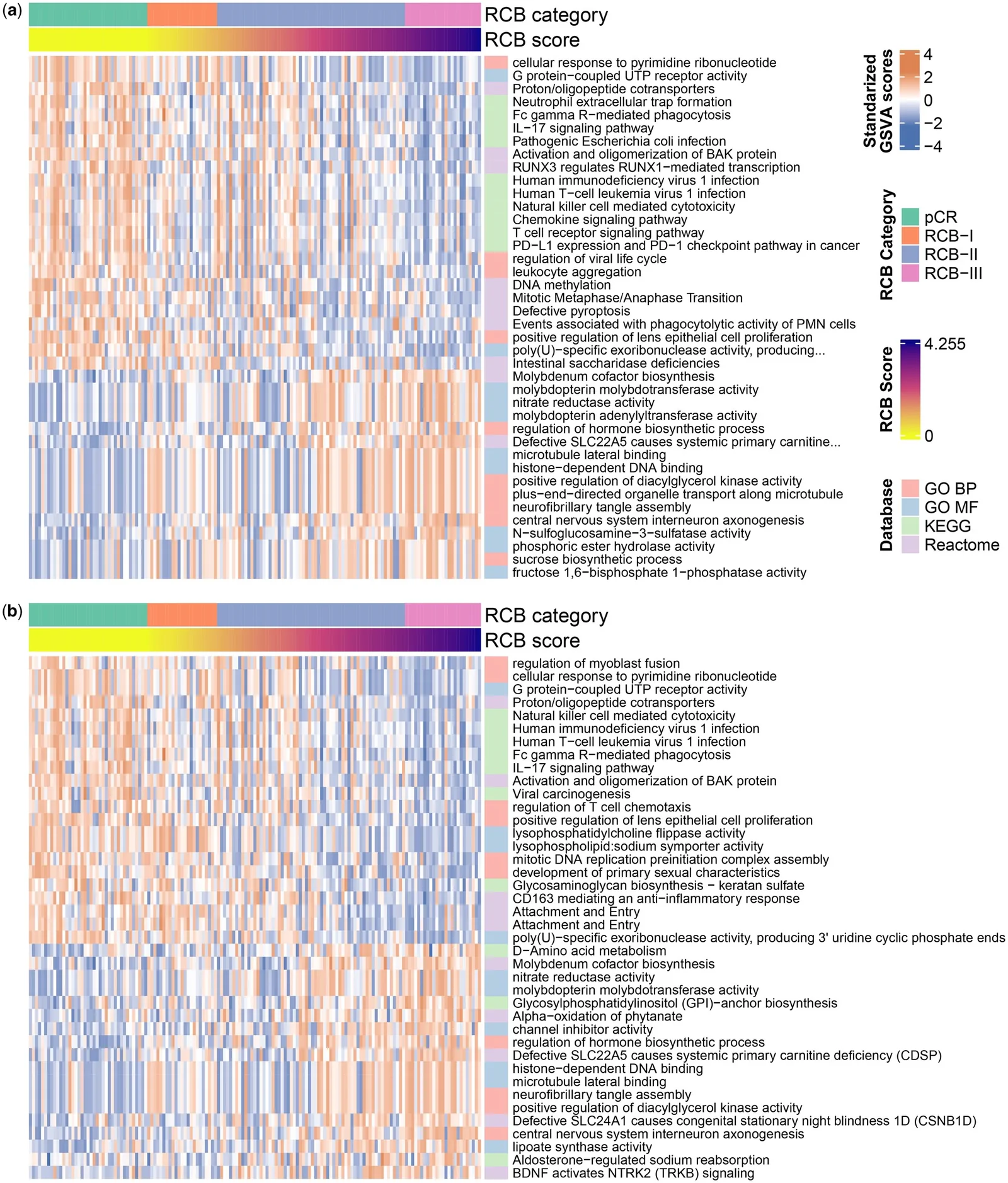
Heatmaps of the most important gene sets for predicting response to neoadjuvant therapy in breast cancer patients. (a) Gene sets selected from the binary pCR/RD predictive models. (b) Gene sets selected from the continuous RCB score prediction. Patients (columns) are ordered by their RCB score. Gene sets (rows) are clustered using hierarchical clustering. Colours in the heatmap represent the calculated GSVA scores for each patient and signature, standardized with Z-score for visualization.

To further demonstrate the application of molecular scores in predicting clinical outcome, even when prediction accuracy is not high, we identified the most important features for these classifications (Tables S4 and S5, available as supplementary data at *Bioinformatics* online). Figure 2 shows the scores of the top 10 gene sets from the obtained models using different databases. Interestingly, the top features included several pathways and biological processes consistent with well-known mechanisms underlying response to chemotherapy. For example, the gene set related to PD-L1 expression and PD-1 checkpoint pathway in cancer showed elevated activities in pCR patients at baseline (Fig. 2a).

This is in line with recent findings that associate the activity of PD-1, an immune checkpoint that inhibits immune response and promotes self-tolerance, and its ligand, PD-L1, with immune cell infiltration into the tumour microenvironment (Buisseret *et al*. 2017). Such immune cell infiltration in tumours has been previously associated with pCR after neoadjuvant therapy in breast cancer patients (Denkert *et al*. 2010, Ignatiadis *et al*. 2012), consistent with our results. Additionally, other immune-related pathways, including natural killer cell mediated cytotoxicity, leucocyte aggregation, and neutrophil extracellular trap formation, also exhibited increased baseline activities in pCR patients, supporting the hypothesis that robust immune activity within the tumour is associated with an improved response to neoadjuvant chemotherapy.

In contrast to those elevated activities, several top gene sets with significant importance in the models showed lower activity scores in pCR patients. For example, the KEGG pathway associated with glycosylphosphatidylinositol (GPI)-anchor biosynthesis showed reduced activities in pCR patients, in contrast to increased activities in patients with extensive residual disease (RCB-III) (Fig. 2b). Overexpression of genes related to GPI functions has been previously linked to increased proliferation and invasion of breast cancer cells (Nagpal *et al*. 2008) and associated with poor survival and adverse clinicopathological characteristics (Zeng *et al*. 2022). Our results suggested that the level of protein binding to the cell surface is associated with the presence of residual disease, potentially due to a more robust and adaptable membrane given a higher GPI level, which leads to reduced susceptibility of tumour cells to chemotherapy.

Regarding the high number of false positives for treatment response in our models, the results are coherent with the original study, which reported that many patients with active tumour immune microenvironment failed to attain pCR (Sammut *et al*. 2022). The authors demonstrated that T cell dysfunction and T cell exclusion were higher in patients with residual disease after treatment. Therefore, the available gene sets definitions permit ML models to detect inactive tumour immune microenvironments associated with a poor treatment response, but other important immune features associated with poor treatment response are not captured with these gene sets, causing a high rate of false positives due to this missing information.

### 3.4 Dissection of heterogeneous pathways in ulcerative colitis

**Figure 3:**
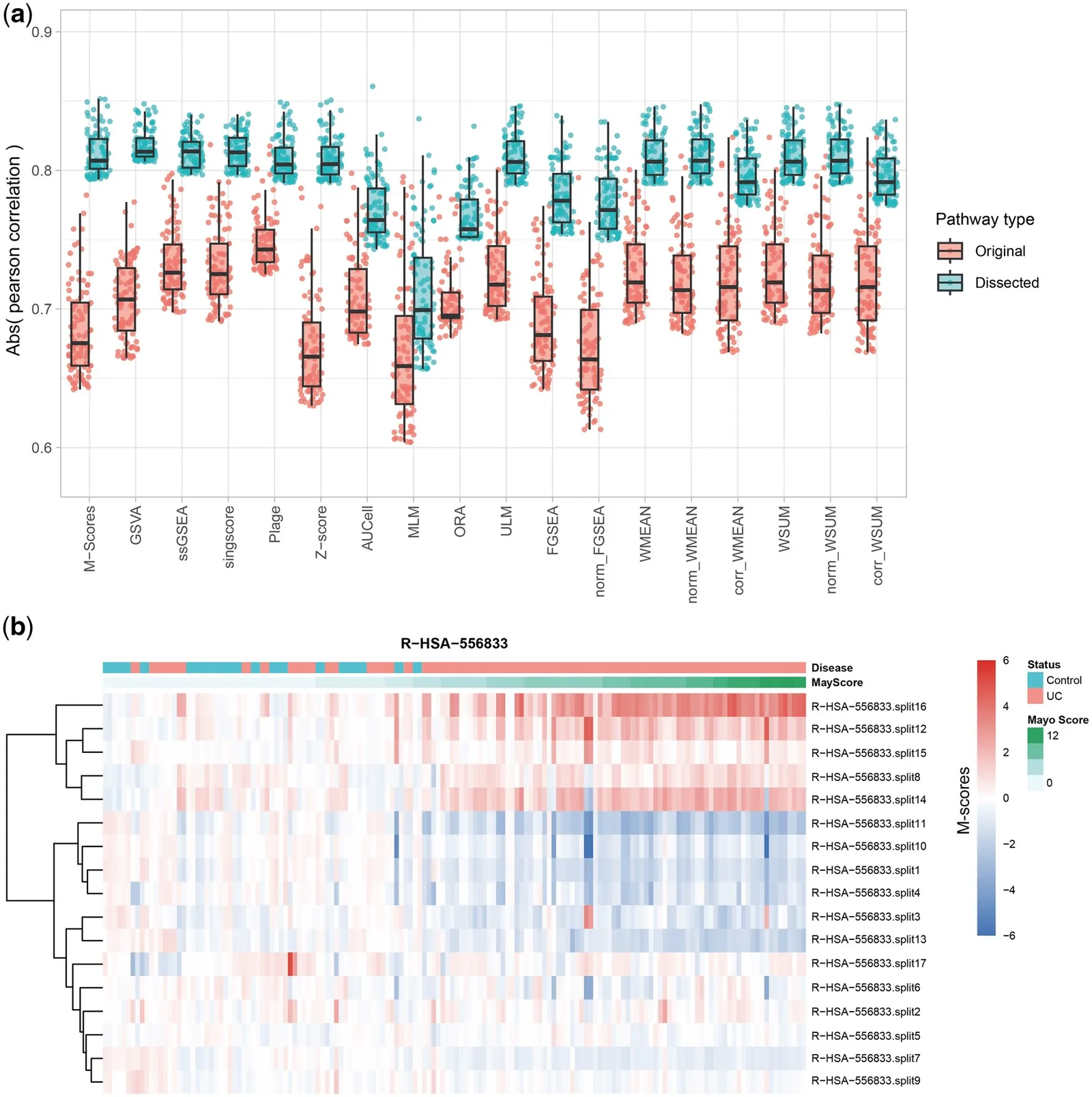
Dissection of Reactome pathways for the study of UC. (a) Boxplot of the absolute Pearson’s correlations between pathway scores and Mayo scores for the top 100 most correlated pathways from the original Reactome database (red) and the subpathways defined by pathMED (blue). *X*-axis contains different scoring methods. For all methods, the mean difference between the top original and dissected pathways was significant (*P*-value < 0.05). (b) Heatmap of the M-scores against the R-HSA-556833 subpathways.

To demonstrate the utility of dissecting general heterogeneous molecular signatures into homogeneous subsignatures, we used gene expression data from UC patients and healthy controls to determine the correlation between clinical disease severities and signature scores when using different signature sets. Specifically, scores were calculated using the original and dissected Reactome pathways, separately. The correlation analysis revealed higher correlations between the signature scores for the top dissected pathways and the clinical severity scores, compared to those observed with scores for the original pathways (Fig. 3a). This trend was consistent across all the scoring methods (Fig. 3a). This finding demonstrated that dividing heterogeneous pathways into granular, coexpressed subpathways can produce molecular scores that more accurately reflect the underlying disease biology. Nevertheless, we compared the capacity to predict the Mayo score of the original and dissected pathways using three different ML algorithms in the pathMED workflow (i.e. RF, KNN and SVM). For most cases, *r* and *R*^2^ were higher using the dissected pathways, while metrics measuring errors (i.e. RMSE, MAE, RMAE and RSE) were lower with the dissected pathways (Fig. S2, available as supplementary data at *Bioinformatics* online).

To further evaluate the level of heterogeneity in the original pathways, we characterised the difference in the signature scores between the original R-HSA-556833 (Metabolism of lipids) pathway and its corresponding 17 subpathways identified with pathMED (Table S6, available as supplementary data at *Bioinformatics* online). We observed that although the signature scores of the original pathway showed a significant negative correlation (−0.5749) with the severity scores, the subpathways exhibited correlations in different directions (i.e. positive or negative), with some lacking significant correlations (Fig. 3b and Table S6, available as supplementary data at *Bioinformatics* online). Among all the subpathways, the split 16 showed the strongest positive correlation with disease severity (*γ* = 0.8288, *P*-value = 1.17·10^−39^). Interestingly, several genes in this subpathway have been previously reported to be positively associated with UC and intestinal inflammation. For example, LPIN1 encodes the lipin-1 phosphatase, which has been associated to the development of colitis-associated tumorigenesis (Meana *et al*. 2018). PTPN13 acts on phosphorylated JAM-A (a tight-junction protein), while increased JAM-A phosphorylation and disrupted barrier function in colonic epithelium were reported in UC patients (Fan *et al*. 2019).

On the other hand, to assess the implications on dissecting a database on the molecular scoring computing time, we measured the necessary time for the dissection process and for molecular scoring with the original and the dissected Reactome pathways (Table S7, available as supplementary data at *Bioinformatics* online). In this dataset, the dissection process only took 27.19 seconds to complete, so it is not a bottleneck for this amount of data. Using the dissected pathways increased the molecular scoring computing time an average of 298.91%, which is coherent with the increase of analysed pathways from 2501 to 10 138 (+305.36%).

Finally, to assess the generalisability of the dissection process across datasets, we used 10 datasets to generate subpathways and, then, we evaluated these subpathways in 4 independent datasets not used during training. Briefly, we assessed whether genes within each subpathway remained more tightly coexpressed compared to the rest of their parent pathway. Supplementary Results contain the description of the methods and results of this analysis. The results demonstrate that the identified subpathways show consistent behaviour across independent cohorts, supporting the robustness, generalisability and stability of the pathway subclustering.

## 4 Conclusions

Molecular scoring provides a robust strategy for addressing challenges related to the utilisation and interpretability of omics data, facilitating its application in precision medicine. Despite existing effort in developing various scoring methods and gene set databases, the process from molecular scoring to downstream applications is complicated using diverse scoring methods and gene set databases, as well as by the lack of robust model training and validation strategies, the use of inadequate performance metrics (e.g. accuracy, F1-score and Receiver Operating Characteristic—Area Under the Curve (Chicco and Jurman 2020, 2023)) or their low generalisability. In this work, we developed pathMED, a comprehensive R/Bioconductor package that integrates 20 molecular scoring methods, 12 preprocessed gene set databases, and 16 ML algorithms, offering a robust framework that covers all necessary steps from scoring to model building.

We demonstrated the applications of pathMED across three different use cases, addressing tasks frequently encountered in precision medicine research. In the multi-omics use case, we demonstrated the potential of pathMED to interoperate across different omics data types. We showed that the models trained with scores derived from transcriptomics to predict tumour from normal lung tissues can be effectively deployed on scores derived from proteomics, suggesting generalisable modelling across different omics data types is achievable using molecular scores.

In the second use case, we demonstrated the application of pathMED in predicting treatment response in breast cancer patients receiving neoadjuvant chemotherapy. Although the predictive power was only moderate when relying solely on tumour transcriptomics data, this approach allowed us to identify response-relevant biological processes. We detected several immune-related pathways, such as PD-1 and natural killer cell activation, associated with robust treatment response, while other pathways such as GPI-anchor biosynthesis related to residual disease, in line with previous reports. We also reported other novel pathways and processes that may be further investigated in the future to understand their implication in breast cancer treatment response.

Lastly, we demonstrated the advantage of incorporating a step to dissect heterogeneous gene sets in revealing disease-relevant signatures. Using transcriptomics data from UC patients and healthy controls, we showed that subpathways derived from the original pathway based on coexpression patterns exhibited stronger associations with disease severity than the original pathways and better predictive capacity. Our characterisation of the lipid metabolism pathway further revealed substantial intra-pathway heterogeneity and identified a subset of genes most strongly associated with disease severity.

In conclusion, pathMED streamlined the process of molecular scoring, signature dissection, and ML modelling, offering an integrated toolkit to simplify applications in precision medicine and the discovery of disease-relevant molecular mechanisms.

## Supplementary Material

btag519_Supplementary_Data

## Data Availability

The datasets were derived from publicly available sources. Multi-omics data are available through the LinkedOmicsKB resource (https://kb.linkedomics.org/). Breast cancer data are available from the European Genome-Phenome Archive (EGA) under accession number EGAS00001004582. Ulcerative colitis data are available from the NCBI Gene Expression Omnibus (GEO) under accession number GSE206171.

## References

[btag519-B1] Ackermann M , Strimmer K. A general modular framework for gene set enrichment analysis. BMC Bioinformatics 2009;10:47. 10.1186/1471-2105-10-47. 19192285 PMC2661051

[btag519-B2] Agrawal A , Balcı H, Hanspers K et al WikiPathways 2024: next generation pathway database. Nucleic Acids Res 2024;52:D679–89. 10.1093/nar/gkad960. 37941138 PMC10767877

[btag519-B3] Aibar S , González-Blas CB, Moerman T et al SCENIC: single-cell regulatory network inference and clustering. Nat Methods 2017;14:1083–6. 10.1038/nmeth.4463. 28991892 PMC5937676

[btag519-B4] Aleksander SA , Balhoff J, Carbon S et al The gene ontology knowledgebase in 2023. Genetics 2023;224:iyad031. 10.1093/genetics/iyad031. 36866529 PMC10158837

[btag519-B5] Ashburner M , Ball CA, Blake JA et al Gene ontology: tool for the unification of biology. Nat Genet 2000;25:25–9. 10.1038/75556. 10802651 PMC3037419

[btag519-B6] Badia-I-Mompel P , Vélez Santiago J, Braunger J et al decoupleR: ensemble of computational methods to infer biological activities from omics data. Bioinform Adv 2022;2:vbac016. 10.1093/bioadv/vbac016. 36699385 PMC9710656

[btag519-B7] Barbie DA , Tamayo P, Boehm JS et al Systematic RNA interference reveals that oncogenic KRAS-driven cancers require TBK1. Nature 2009;462:108–12. 10.1038/nature08460. 19847166 PMC2783335

[btag519-B8] Breiman L. Random forests. Mach Learn 2001;45:5–32. 10.1023/A:1010933404324.

[btag519-B9] Breiman L , Friedman JH, Olshen RA et al Classification and Regression Trees. New York: Chapman and Hall/CRC, 1984. 10.1201/9781315139470.

[btag519-B10] Buisseret L , Garaud S, Wind A et al Tumor-infiltrating lymphocyte composition, organization and PD-1/ PD-L1 expression are linked in breast cancer. Oncoimmunology 2017;6:e1257452. 10.1080/2162402X.2016.1257452. 28197375 PMC5283629

[btag519-B11] Chaussabel D , Quinn C, Shen J et al A modular analysis framework for blood genomics studies: application to systemic lupus erythematosus. Immunity 2008;29:150–64. 10.1016/j.immuni.2008.05.012. 18631455 PMC2727981

[btag519-B12] Chen T , Guestrin C. XGBoost: A scalable tree boosting system. In: *Proceedings of the 22nd ACM SIGKDD International Conference on Knowledge Discovery and Data Mining (KDD '16)*. New York, NY, USA: Association for Computing Machinery (ACM), 2016, 785–94. 10.1145/2939672.2939785

[btag519-B13] Chen Y , Chen L, Lun ATL et al edgeR v4: powerful differential analysis of sequencing data with expanded functionality and improved support for small counts and larger datasets. Nucleic Acids Res 2025;53:gkaf018. 10.1093/nar/gkaf018. 39844453 PMC11754124

[btag519-B14] Chen YJ , Roumeliotis TI, Chang YH et al Proteogenomics of non-smoking lung cancer in east asia delineates molecular signatures of pathogenesis and progression. Cell 2020;182:226–44.e17. 10.1016/j.cell.2020.06.012. 32649875

[btag519-B15] Chicco D , Jurman G. The advantages of the Matthews correlation coefficient (MCC) over F1 score and accuracy in binary classification evaluation. BMC Genomics 2020;21:6. 10.1186/s12864-019-6413-7. 31898477 PMC6941312

[btag519-B16] Chicco D , Jurman G. The Matthews correlation coefficient (MCC) should replace the ROC AUC as the standard metric for assessing binary classification. BioData Min 2023;16:4. 10.1186/s13040-023-00322-4. 36800973 PMC9938573

[btag519-B17] Cortes C , Vapnik V. Support-vector networks. Mach Learn 1995;20:273–97. 10.1007/BF00994018.

[btag519-B18] Cox DR. The regression analysis of binary sequences. J R Stat Soc Ser B Methodol 1958;20:215–32.

[btag519-B19] Dan Foresee F , Hagan MT. Gauss-Newton approximation to Bayesian learning. Proc Int Conf Neural Netw ICNN97 1997;3:1930–5. 10.1109/ICNN.1997.614194.

[btag519-B20] Davis AP , Wiegers TC, Sciaky D et al Comparative Toxicogenomics Database’s 20th anniversary: update 2025. Nucleic Acids Res 2025;53:D1328–34. 10.1093/nar/gkae883. 39385618 PMC11701581

[btag519-B21] Denkert C , Loibl S, Noske A et al Tumor-associated lymphocytes as an independent predictor of response to neoadjuvant chemotherapy in breast cancer. J Clin Oncol 2010;28:105–13. 10.1200/JCO.2009.23.7370. 19917869

[btag519-B22] Efron B , Hastie T, Johnstone I et al Least angle regression. Ann Statist 2004;32:407–99. 10.1214/009053604000000067.

[btag519-B23] Fan S , Weight CM, Luissint AC et al Role of JAM-A tyrosine phosphorylation in epithelial barrier dysfunction during intestinal inflammation. Mol Biol Cell 2019;30:566–78. 10.1091/mbc.E18-08-0531. 30625033 PMC6589701

[btag519-B24] Foroutan M , Bhuva DD, Lyu R et al Single sample scoring of molecular phenotypes. BMC Bioinformatics 2018;19:404. 10.1186/s12859-018-2435-4. 30400809 PMC6219008

[btag519-B25] Garcia-Moreno A , López-Domínguez R, Villatoro-García JA et al Functional enrichment analysis of regulatory elements. Biomedicines 2022;10:590. 10.3390/biomedicines10030590. PMC8945021 35327392

[btag519-B26] Gargano MA , Matentzoglu N, Coleman B et al The Human Phenotype Ontology in 2024: phenotypes around the world. Nucleic Acids Res 2024;52:D1333–D1346. 10.1093/nar/gkad1005. 37953324 PMC10767975

[btag519-B27] Hänzelmann S , Castelo R, Guinney J. GSVA: gene set variation analysis for microarray and RNA-Seq data. BMC Bioinformatics 2013;14:7. 10.1186/1471-2105-14-7. 23323831 PMC3618321

[btag519-B28] Hejblum BP , Skinner J, Thiébaut R. Time-course gene set analysis for longitudinal gene expression data. PLoS Comput Biol 2015;11:e1004310. 10.1371/journal.pcbi.1004310. 26111374 PMC4482329

[btag519-B29] Hubbard EL , Bachali P, Kingsmore KM et al Analysis of transcriptomic features reveals molecular endotypes of SLE with clinical implications. Genome Med 2023;15:84. 10.1186/s13073-023-01237-9. 37845772 PMC10578040

[btag519-B30] Ignatiadis M , Singhal SK, Desmedt C et al Gene modules and response to neoadjuvant chemotherapy in breast cancer subtypes: a pooled analysis. J Clin Oncol 2012;30:1996–2004. 10.1200/JCO.2011.39.5624. 22508827

[btag519-B31] Kanehisa M , Goto S. KEGG: Kyoto Encyclopedia of Genes and Genomes. Nucleic Acids Res 2000;28:27–30. 10.1093/nar/28.1.27. 10592173 PMC102409

[btag519-B32] Karczewski KJ , Snyder MP. Integrative omics for health and disease. Nat Rev Genet 2018;19:299–310. 10.1038/nrg.2018.4. 29479082 PMC5990367

[btag519-B33] Keenan AB , Jenkins SL, Jagodnik KM et al The library of integrated network-based cellular signatures NIH program: system-level cataloging of human cells response to perturbations. Cell Syst 2018;6:13–24. 10.1016/j.cels.2017.11.001. 29199020 PMC5799026

[btag519-B34] Kenney JF , Keeping ES. Linear Regression and Correlation. 3rd ed. Princeton, NJ: Van Nostrand, 1962.

[btag519-B35] Kim H , Jesus AA, de Brooks SR et al Development of a validated interferon score using NanoString technology. J Interferon Cytokine Res 2018;38:171–85. 10.1089/jir.2017.0127. 29638206 PMC5963606

[btag519-B36] Korotkevich G , Sukhov V, Budin N et al Fast gene set enrichment analysis. bioRxiv, 10.1101/060012, 1 Jan 2021, 060012.

[btag519-B37] Kuhn M. Building predictive models in R using the caret package, articles. J Stat Soft 2008;28:1–26. 10.18637/jss.v028.i05.

[btag519-B38] Lee E , Chuang HY, Kim JW et al Inferring pathway activity toward precise disease classification. PLoS Comput Biol 2008;4:e1000217. 10.1371/journal.pcbi.1000217. 18989396 PMC2563693

[btag519-B39] Li S , Rouphael N, Duraisingham S et al Molecular signatures of antibody responses derived from a systems biology study of five human vaccines. Nat Immunol 2014;15:195–204. 10.1038/ni.2789. 24336226 PMC3946932

[btag519-B40] MacKay DJC. Bayesian interpolation. Neural Comput 1992;4:415–47. 10.1162/neco.1992.4.3.415.

[btag519-B41] McCullagh P. Generalized Linear Models. 2nd ed. London: Chapman and Hall; 1989. 10.1201/9780203753736.

[btag519-B42] Meana C , García-Rostán G, Peña L et al The phosphatidic acid phosphatase lipin-1 facilitates inflammation-driven Colon carcinogenesis. JCI Insight 2018;3:e97506. 10.1172/jci.insight.97506. PMC6237220 30232275

[btag519-B43] Milacic M , Beavers D, Conley P et al The reactome pathway knowledgebase 2024. Nucleic Acids Res 2024;52:D672–8. 10.1093/nar/gkad1025. 37941124 PMC10767911

[btag519-B44] Mucherino A , Papajorgji PJ, Pardalos PM. k-nearest neighbor classification. In: Mucherino A, Papajorgji PJ, Pardalos PM (eds.), Data Mining in Agriculture. New York, NY: Springer New York, 2009, 83–106. 10.1007/978-0-387-88615-2_4.

[btag519-B45] Nagpal JK , Dasgupta S, Jadallah S et al Profiling the expression pattern of GPI transamidase complex subunits in human cancer. Mod Pathol 2008;21:979–91. 10.1038/modpathol.2008.76. 18487995 PMC3082921

[btag519-B46] Pavlidis S , Monast C, Loza MJ et al I_MDS: an inflammatory bowel disease molecular activity score to classify patients with differing disease-driving pathways and therapeutic response to anti-TNF treatment. PLoS Comput Biol 2019;15:e1006951. 10.1371/journal.pcbi.1006951. 31039157 PMC6510457

[btag519-B47] Piñero J , Ramírez-Anguita JM, Saüch-Pitarch J et al The DisGeNET knowledge platform for disease genomics: 2019 update. Nucleic Acids Res 2020;48:D845–D855. 10.1093/nar/gkz1021. 31680165 PMC7145631

[btag519-B48] Ripley BD. Pattern Recognition and Neural Networks. Cambridge: Cambridge University Press, 1996. 10.1017/CBO9780511812651.

[btag519-B49] Robinson MD , Oshlack A. A scaling normalization method for differential expression analysis of RNA-seq data. Genome Biol 2010;11:R25. 10.1186/gb-2010-11-3-r25. 20196867 PMC2864565

[btag519-B50] Sammut SJ , Crispin-Ortuzar M, Chin SF et al Multi-omic machine learning predictor of breast cancer therapy response. Nature 2022;601:623–9. 10.1038/s41586-021-04278-5. 34875674 PMC8791834

[btag519-B51] Satpathy S , Clark NM, Chen YJ et al Integrative analysis of lung adenocarcinoma across diverse ethnicities and exposures. Cancer Cell 2025;43:1731–57.e17. 10.1016/j.ccell.2025.07.011. 40749670 PMC12393171

[btag519-B52] Schapire RE. Explaining AdaBoost. In: Schölkopf B, Luo Z, Vovk V (eds.), Empirical Inference: Festschrift in Honor of Vladimir N. Vapnik. Berlin, Heidelberg: Springer Berlin Heidelberg, 2013, 37–52. 10.1007/978-3-642-41136-6_5.

[btag519-B53] Schroeder KW , Tremaine WJ, Ilstrup DM. Coated oral 5-aminosalicylic acid therapy for mildly to moderately active ulcerative colitis. N Engl J Med 1987;317:1625–9. 10.1056/NEJM198712243172603. 3317057

[btag519-B54] Soendergaard C , Kvist PH, Thygesen P et al Characterization of growth hormone resistance in experimental and ulcerative colitis. Int J Mol Sci 2017;18:2046. 10.3390/ijms18102046. 28946616 PMC5666728

[btag519-B55] Teschendorff AE , Wang N. Improved detection of tumor suppressor events in single-cell RNA-Seq data. Npj Genomic Med 2020;5:43. 10.1038/s41525-020-00151-y. PMC7541488 33083012

[btag519-B56] Tomfohr J , Lu J, Kepler TB. Pathway level analysis of gene expression using singular value decomposition. BMC Bioinform 2005;6:225. 10.1186/1471-2105-6-225. PMC1261155 16156896

[btag519-B57] Toro-Domínguez D , Martorell-Marugán J, Martinez-Bueno M et al Scoring personalized molecular portraits identify Systemic Lupus Erythematosus subtypes and predict individualized drug responses, symptomatology and disease progression. Brief Bioinform 2022;23:bbac332. 10.1093/bib/bbac332. 35947992 PMC9487588

[btag519-B58] Toro-Domínguez D , Wang C, Ellson-Lancho I et al Benchmarking single-sample gene set scoring methods for application in precision medicine. Brief Bioinform 2025;26:bbaf684. 10.1093/bib/bbaf684. 41405962 PMC12710473

[btag519-B59] Tutz G , Binder H. Generalized additive modeling with implicit variable selection by likelihood-based boosting. Biometrics 2006;62:961–71. 10.1111/j.1541-0420.2006.00578.x. 17156269

[btag519-B60] Whirl-Carrillo M , Huddart R, Gong L et al An evidence-based framework for evaluating pharmacogenomics knowledge for personalized medicine. Clin Pharmacol Ther 2021;110:563–72. 10.1002/cpt.2350. 34216021 PMC8457105

[btag519-B61] Zeng J , Yi J, Tan S et al GPI: an indicator for immune infiltrates and prognosis of human breast cancer from a comprehensive analysis. Front Endocrinol 2022;13:995972. 10.3389/fendo.2022.995972. PMC9554491 36246907

[btag519-B62] Zhang H. The optimality of Naive Bayes. In: *Proceedings of the Seventeenth International Florida Artificial Intelligence Research Society Conference*, Menlo Park, CA, USA: AAAI Press, 2004, 562–7.

[btag519-B63] Zou H , Hastie T. Regularization and variable selection via the elastic net. J R Stat Soc Ser B 2005;67:301–20.

[btag519-B64] Zyla J , Marczyk M, Domaszewska T et al Gene set enrichment for reproducible science: comparison of CERNO and eight other algorithms. Bioinformatics 2019;35:5146–54. 10.1093/bioinformatics/btz447. 31165139 PMC6954644

