# Supplementary material for: PathMED: an R toolkit for single-sample molecular scoring and machine learning with omics data
Source: Bioinformatics. 2026 Jul 24;42(8):btag519. doi: 10.1093/bioinformatics/btag519 (PMC13430661; doi:10.1093/bioinformatics/btag519)
Supplement: btag519_Supplementary_Data [file btag519_supplementary_data.zip › Supplementary Figures.pdf]

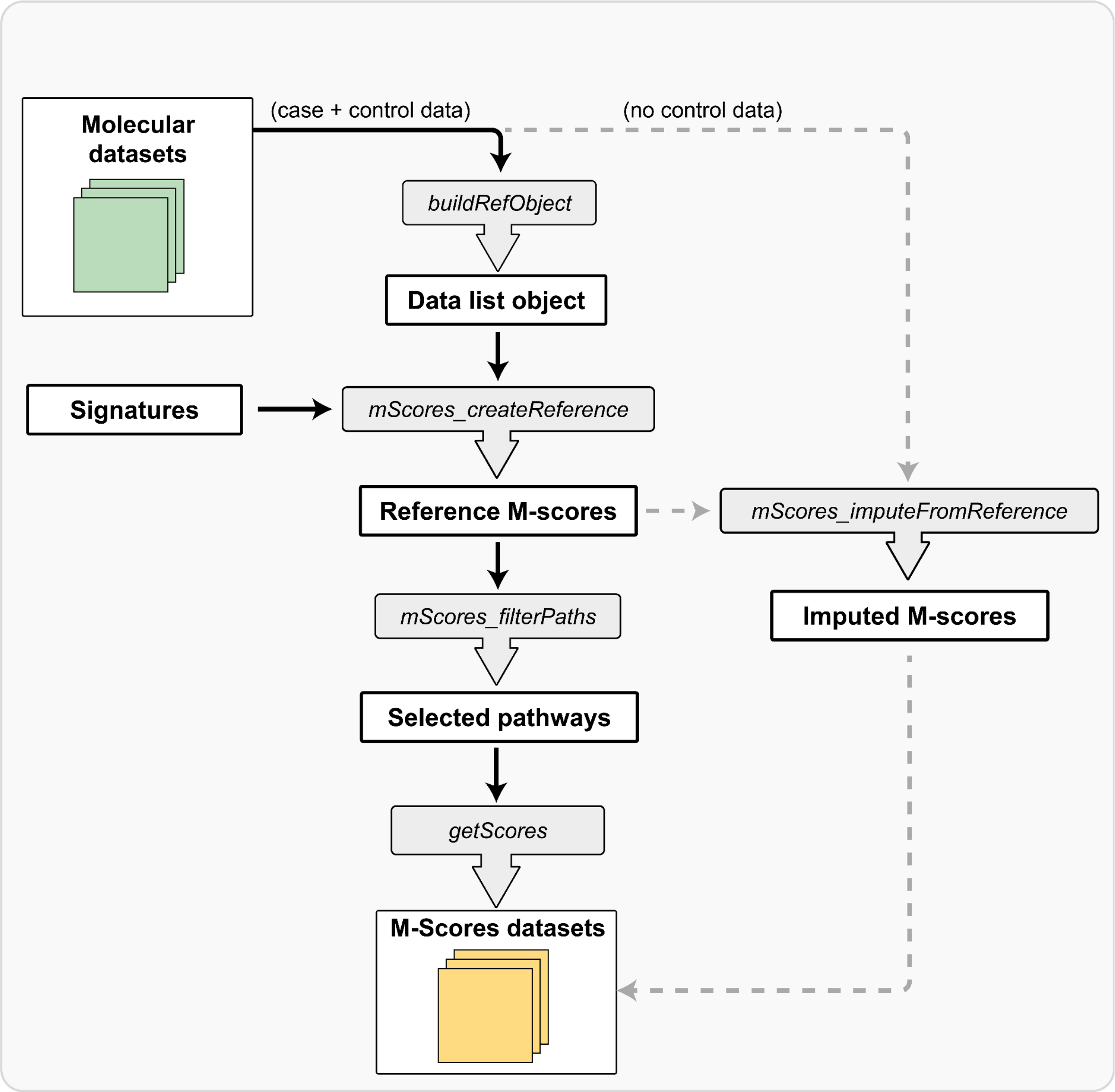

Supplementary Figure 1. Pipeline to create a reference for the M-Score method. Gray boxes represent pathMED functions.

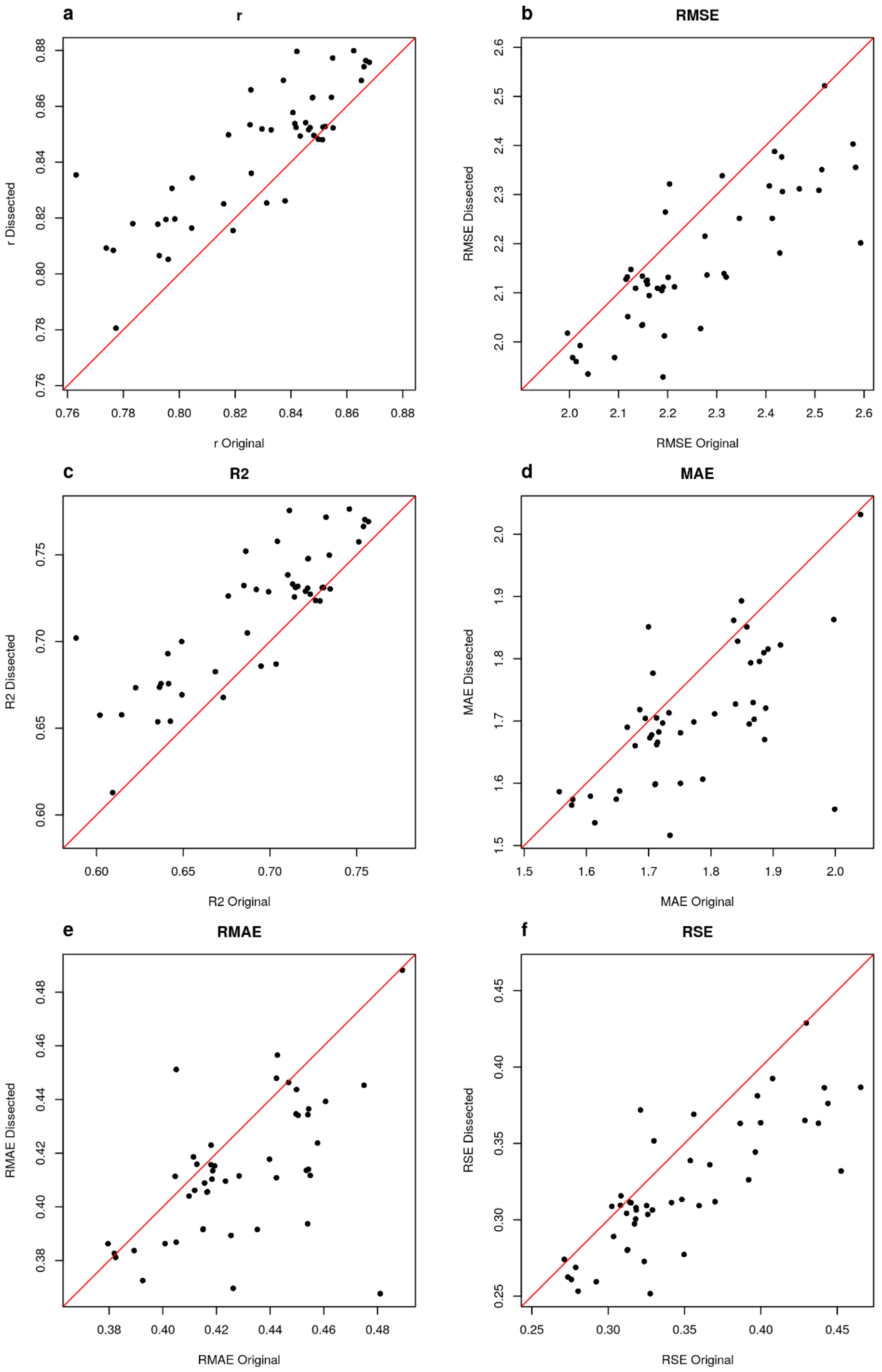

Supplementary Figure 2. Performance metrics for the Mayo score prediction using the original (X-axis) and the dissected (Y-axis) Reactome database. Each point corresponds to the outer CV performance for a scoring method and ML algorithm combination.
