# Supplementary material for: PathMED: an R toolkit for single-sample molecular scoring and machine learning with omics data
Source: Bioinformatics. 2026 Jul 24;42(8):btag519. doi: 10.1093/bioinformatics/btag519 (PMC13430661; doi:10.1093/bioinformatics/btag519)
Supplement: btag519_Supplementary_Data [file btag519_supplementary_data.zip › Supplementary_Results.pdf]

Supplementary Results

External validation of subpathway reproducibility

To evaluate the reproducibility and generalizability of the subpathways generated by dissectDB function, an external validation analysis was performed using independent datasets not involved in subpathway construction.

Briefly, Reactome pathways were dissected into coexpressed subpathways using the pathMED framework and ten independent systemic lupus erythematosus (SLE) transcriptomic datasets previously described (Toro-Domínguez *et al.* 2026). Pathway dissection was performed using dissectDB function based on gene coexpression patterns across datasets.

To assess whether the resulting subpathways represented reproducible expression modules rather than dataset-specific partitions, four independent validation datasets, not used during subpathway generation, were analyzed. For each validation dataset,pairwise Pearson correlations were calculated between all genes belonging to a given parent Reactome pathway. Correlation matrices were transformed into distance matrices using the metric: $d=1-r$, where r denotes the Pearson correlation coefficient between gene expression profiles. For each subpathway, genes were assigned to two groups: (i) genes belonging to the subpathway and (ii) the remaining genes from the original parent pathway. The average silhouette width was then calculated for genes belonging to the subpathway, providing a quantitative measure of how well the genes within the subpathway remain separated from the rest of the pathway in independent cohorts.

To generate a null distribution, 1,000 random gene subsets of identical size were sampled from the same parent pathway and evaluated using the same procedure. The mean silhouette value obtained from these random subsets was used as the reference expectation for each subpathway. Average silhouette values were subsequently summarized across the four validation datasets for both observed and random subpathways.

Across validation cohorts, genes belonging to pathMED-derived subpathways consistently exhibited higher silhouette values than randomly generated gene subsets of identical size sampled from the same parent pathway (Figure Supp1). This pattern was observed throughout the entire range of evaluated subpathways and was highly consistent across validation datasets.

The observed silhouette values remained substantially above the random expectation for most subpathways, indicating that genes assigned to the same subpathway maintained coherent expression patterns in independent cohorts. In contrast, random gene subsets generated from the same pathways produced silhouette values close to zero, consistent with the absence of meaningful internal structure.

These results demonstrate that the gene groupings identified during pathway dissection are reproducible across independent datasets and do not simply reflect dataset-specific clustering artifacts. Instead, the identified subpathways represent stable coexpression modules that can be consistently recovered in

external cohorts, supporting the robustness and generalizability of the pathMED pathway dissection strategy.

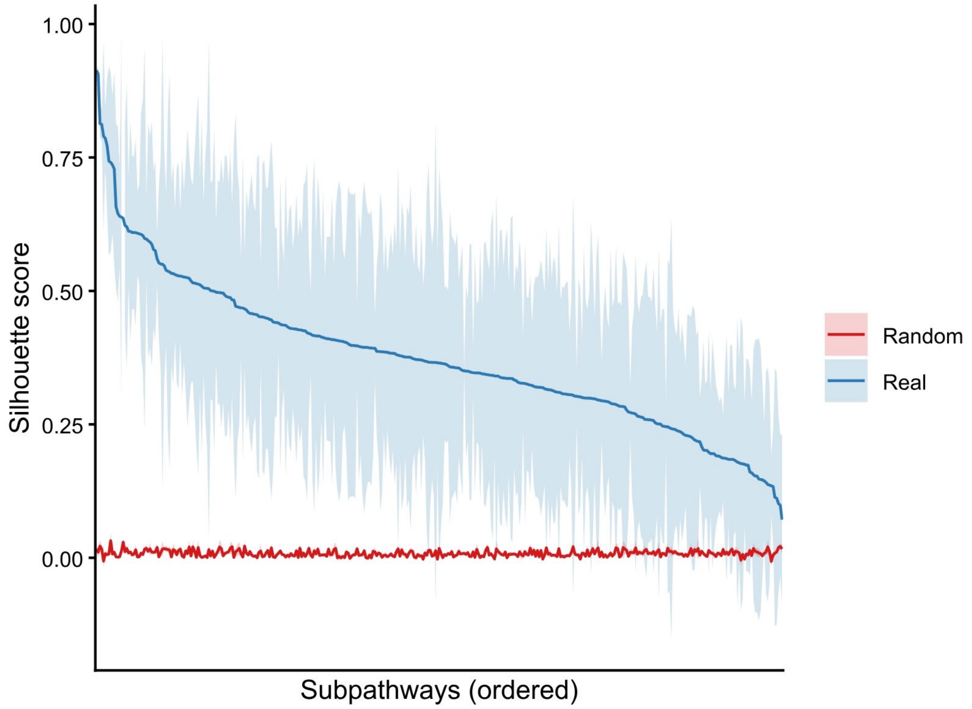

Figure Supp1. External validation of pathMED-derived subpathways. Mean silhouette values (± SD across four independent validation datasets) for subpathways generated using ten training datasets. Subpathways are ordered according to their average observed silhouette value. Blue lines represent the observed subpathways generated by dissectDB function, whereas red lines correspond to random gene subsets of identical size sampled from the same parent pathway

Supplementary References

Toro-Domínguez D, Wang C, Ellson-Lancho I *et al.* SLE-diseaseome: a comprehensive meta-collection of systemic lupus erythematosus relevant functional pathways. *Bioinforma Adv* 2026;**6**(1):vbag061. https://doi.org/10.1093/bioadv/vbag061.
